# The humanistic, economic and societal burden of Herpes Zoster in Europe: a critical review

**DOI:** 10.1186/s12889-015-1514-y

**Published:** 2015-02-27

**Authors:** Adam Gater, Mathieu Uhart, Rachael McCool, Emmanuelle Préaud

**Affiliations:** Adelphi Values, Adelphi Mill, Grimshaw Lane, Bollington, Cheshire SK10 5JB UK; Sanofi Pasteur MSD, 162 avenue Jean Jaurès, Lyon, France

## Abstract

**Background:**

Herpes zoster (HZ) or “shingles” is common in persons aged 50 years or over. HZ is characterised by a painful dermatological rash which typically resolves in approximately one month. Persistent pain for months or years after rash onset, however, is a common complication of HZ; referred to as post-herpetic neuralgia (PHN). Both HZ and PHN have a significant impact on patients’ lives, with considerable implications for healthcare systems and wider society. The aim of the present review is to provide comprehensive documentation and critical appraisal of published data concerning the humanistic, economic and societal burden of HZ in Europe.

**Methods:**

Systematic literature searches were conducted in Medline, EMBASE, PsycINFO, EconLit, HEED and CRD databases. Searches were conducted in July 2014 and restricted to articles published in the past 20 years. Articles were selected for full review by two independent researchers in accordance with predefined eligibility criteria.

**Results:**

From a review of 1619 abstracts, 53 eligible articles, were identified which reported data concerning healthcare resource use (n = 38), direct costs (n = 20), indirect costs (n = 16), total costs (n = 10) and impact on health-related quality of life (HRQoL) (n = 21). Findings highlight that PHN is associated with greater impairments in HRQoL and higher costs of management than HZ. For both HZ and PHN, pain severity is a significant predictor of impact on individuals, healthcare systems and society. While the incidence of HZ and PHN increase with age, age does not appear to be a key driver of overall costs for HZ and PHN. Specifically, while direct costs (e.g. GP, specialists, medications, hospitalisations) tend to be higher for older patients, indirect costs (e.g. work time missed) are higher for younger patients.

**Conclusions:**

Available evidence highlights that HZ and PHN result in significant humanistic and economic burden for patients, healthcare systems and wider societies. A tendency to focus upon healthcare resource use and the direct costs of management at the expense of other impacts (e.g. informal caregivers and formal social care) may result in an underestimation of the true burden of HZ and PHN.

## Background

Herpes zoster (HZ), also known as shingles, is caused by the reactivation of the varicella zoster virus (VZV). Primary infection with VZV usually occurs during childhood, manifesting as chickenpox, after which the virus lies dormant [1]. The great majority (95%) [2] of the population hosts VZV and approximately one in four persons will develop HZ in their lifetime through reactivation, leading to an estimated 1.7 million episodes in Europe annually [3]. VZV reactivation is primarily related to an age-related decline in VZV-specific immunity; it is well documented that the incidence of HZ increases with age, with the majority of cases occurring in patients over 50 years of age [4].

HZ is characterized by a painful dermatomal rash, which most commonly presents on the trunk and lumbar regions. HZ, however, may present along any nerve including the ophthalmic division of the trigeminal nerve where it can affect the eye. Herpes Zoster Ophthalmicus (HZO) occurs in 10-20% of all HZ cases and in some cases can lead to ocular complications [5].

The dermatological rash and pain associated with HZ typically resolves within one month of presentation [4]; however, long-term complications can arise. Post-herpetic neuralgia (PHN) is the most common and debilitating complication of HZ, occurring in approximately 10-20% of all sufferers [6-8] and as many as 50% of those aged 85 years and over [9]. PHN is a neuropathic syndrome manifesting as on-going pain along the nerves in the area of the prior HZ rash and typically involves one or more of the following: spontaneous aching or burning; paroxysmal shooting pains; allodynia, and hyperalgesia [9,10]. There is no consensus regarding the definition of PHN; however, PHN is often defined as pain persisting for 90 days from HZ rash onset [4,6,10,11]. While HZ is an acute experience that typically resolves within one month, in the majority of sufferers, PHN persists for at least 6 months with some patients experiencing PHN for years [12-15]. As expected given the relationship to HZ, the incidence of PHN increases markedly with age [16-18]. The incidence of PHN is also linked to the severity of pain experienced during the prior HZ episode (among other predictors such as the severity of the dermatomal rash and prodromal symptoms) [19], with those patients experiencing the highest levels of pain during HZ presentation being most at risk of developing PHN [20].

As conditions primarily affecting older persons, many patients with HZ and PHN may already experience significant deficits in health status. Findings from a number of articles indicate that the pain and resulting discomfort associated with HZ and PHN can have a substantial negative and widespread impact on patients’ health-related quality of life (HRQoL) and patients’ ability to engage in activities of daily living [7,20-25]. In addition to the burden encountered by the individual with HZ, the condition is also associated with considerable economic burden. For example, considerable costs to healthcare systems [26-31] arise from the care provision for HZ and PHN patients, including visits to primary care (general practitioner) and outpatient secondary/tertiary care (specialist pain clinics and ophthalmologists), inpatient visits (hospitalisations) and prescription costs. Furthermore, HZ and PHN are also associated with significant indirect costs, primarily in terms of loss of productivity for younger patients [7,21,23,24,28,32]. Finally, as conditions occurring in retired and older patients, HZ and PHN can also have significant consequences for the caregivers of patients including partners, relatives, and friends of people with the conditions [7]. Such details, however, may often go unreported and are not often communicated in the research literature.

Evidence for the efficacy of a VZV vaccine in the prevention of HZ and PHN episodes and attenuation of the severity of HZ and PHN episodes and associated impact has been documented in recent years [11,33]. In order to understand the value of preventative strategies, a complete understanding of the impact of HZ and PHN is required. While a significant body of literature has sought to determine the impact of HZ and PHN on patients, healthcare systems and wider society within EU countries, to date, no comprehensive review of these studies has been conducted. The aim of this review, therefore, is to provide a comprehensive and holistic overview of the humanistic, economic and societal burden of HZ and PHN in Europe as reported within the published peer-reviewed literature.

## Methods

Published peer-reviewed articles were identified via systematic searches conducted in the following biomedical and economic databases: EMBASE, Medline, PsycINFO, EconLit, Health Economic Evaluations Database (HEED) and the Centre for Reviews and Dissemination databases at the University of York. Searches and reviews were conducted in accordance with the Cochrane systematic review guidelines [34]. A range of disease-related, economic, societal and HRQoL-related keywords was utilized to capture the humanistic, economic and societal burden of HZ and PHN (see Table 1). Searches were conducted in July 2014 and were restricted to articles published in the last 20 years. Although data relating to the EU was the primary focus of this review, no limits regarding study country or article language were implemented during the search stage (so to not exclude potentially relevant articles). Instead, articles focused on the EU were prioritized as part of the article selection process.

**Table 1:** **Search terms**

**Databases searched**	**Disease search terms**	**Economic, humanistic and societal burden search terms**
**• Medline**	“Herpes Zoster” OR “Shingles” OR “Post-herpetic Neuralgia” OR “Post herpetic Neuralgia” OR “Postherpetic Neuralgia” OR “PHN”	“Burden” OR “Humanistic” OR “quality of life” OR “QOL” OR “HRQOL” OR “cost” OR “economic” OR “resource” OR “society” OR “societal” OR “family” OR “relatives” OR “Caregiver” OR “care$” OR “activities of daily living” OR “ADLS” OR “daily activities” OR “cost of illness” OR “autonomy” OR “hospitalisation” OR “hospitalization”
**• Embase**		
**• PscyINFO**		
**• EconLit.**		
**• Health Economic Evaluations Database (HEED)**		
**• Centre for Reviews and Dissemination databases:**		
**- Database of Abstracts of Reviews of Effects (DARE),**		
**- Health Technology Assessment (HTA)**		
**- NHS Economic Evaluation Database (NHS EED)**		

In accordance with Cochrane review guidelines [35], all abstracts were reviewed by two researchers independently according to formal inclusion/exclusion criteria (Table 2). For inclusion in the review, abstracts were required present information specific to adult patients in the EU and to contain information pertaining to the key search terms (see Table 1). In instances were aforementioned details were not clear from the abstract, full text articles were obtained to confirm whether the article should be included/excluded. Real world data, observational studies, effectiveness studies, qualitative studies, economic evaluations and cost studies were all considered relevant for review. The final list of abstracts and articles for full in-depth review was agreed following consensus between the authors.

**Table 2:** **Study Inclusion/exclusion criteria**

	**Inclusion criteria**	**Exclusion criteria**
**Country setting**	*Present data from a country in the EU or EU in general*	*Only present data from a non-EU country. No data specific to the EU.*
**Population**	*Adults with HZ or PHN*	*Children*
		*Focus mainly on chicken pox*
		*Immunodepressed patients only (*e.g. *HIV patients)*
	*Studies that contain information on key search terms (inc. QoL, burden, productivity)*	*Studies that do NOT contain information on key search terms*
**Study design**	*Real world data, observational studies, effectiveness, qualitative research and cost studies*	*Clinical trials of efficacy and safety of treatments presenting QoL data as secondary endpoint only*
		*Clinical trials of Zostavax*
		*Focus mainly on epidemiological evidence (*e.g. *incidence, prevalence etc.)*
**Publication type**	*For conference abstracts, exclusion if manuscript corresponding to this abstract and presenting equivalent information is already published*	

Data extraction tables were developed to accurately record information from the articles chosen for review. Of importance was information related to the study aims, design and outcomes of interest, sample characteristics, country of study, resource use data, cost data (direct and indirect) and any patient-reported impacts of HZ and PHN. All referenced costs are provided in Euros and have been adjusted to the same price year (2015) based on annual average rate of change (%) in the Harmonised Index of Consumer Prices [36].

## Results

### Search results

**Figure 1:**
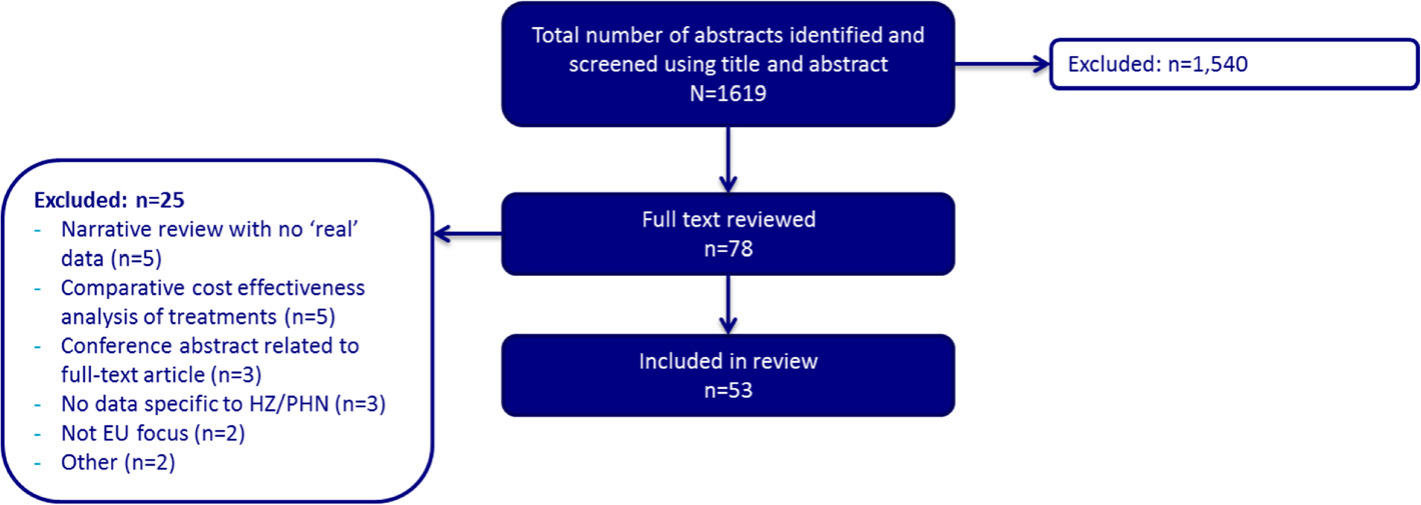
**Overview of study selection for review.**

Figure 1 provides an overview of the article selection process. A total of 1619 titles and abstracts were reviewed against the inclusion/exclusion criteria, resulting in 78 EU based articles for full text review. A further 25 articles were excluded following full text review, leaving a total of 53 studies to be included in this review.

### Overview of selected articles

A summary of the 53 articles selected for inclusion in the review (including study countries, population, data presented) is provided in Table 3.

**Table 3:** **Overview of included studies**

**Author and year**	**Country perspective**	**Population**					**Healthcare utilisation**	**Economic data**			**HRQoL**
		**HZ**	**HZ and PHN**	**PHN1**	**PHN3**	**PHN Other/Undefined**		**Direct costs**	**Indirect costs**	**Total costs**	
Annemans et al. (2010) [26]	Belgium	✓	X	✓	✓	X	✓	✓	✓	✓	X
Bayas *et al.* (2011) [37]	Spain Catalonia	X	✓	X	X	X	✓	✓	X	X	X
Berrut, G. and C. Baptiste (2013) [38]	France	✓	X	X	X	X	✓	X	X	X	X
Blein, C., et al. (2013) [39]	France	✓	X	X	X	X	✓	X	X	X	X
Bilcke *et al.* (2012) [40]	Belgium	X	✓	X	✓	X	✓	✓	✓	X	X
Bouhassira *et al.* (2012) [15]	France	✓	✓	X	✓	X	X	X	X	X	✓
Bresse, X., et al. (2013) [41]	France	✓	X	X	✓	X	✓	X	X	X	X
Bricout, H., et al. (2013) [42]	Italy	✓	X	X	X	X	X	X	✓	X	✓
Bricout, H., et al. (2013) [42]	Italy	✓	X	X	✓	X	✓	X	X	X	✓
Brisson *et al.* (2003) [43]	England & Wales	X	✓	X	X	✓	✓	✓	✓	✓	X
Castro-Lopes, J. M., et al. (2013) [44]	Portugal	X	X	X	X	✓	✓	X	X	X	✓
Cebrian-Cuenca *et al.* (2011) [27]	Spain, Valencia	✓	X	✓	✓	X	✓	✓	✓	✓	X
Chidiac *et al.* (2001) [21]	France	✓	✓	X	X	✓	✓	X	X	X	✓
De Juanes *et al.* (2011) [45]	Spain Madrid	X	✓	X	X	✓	✓	✓	X	X	X
Di Legami *et al.* (2007) [46]	Italy – Piemonte	✓	X	X	X	✓	✓	✓	X	X	X
Edmunds *et al.* (2001) [47]	England and Wales	X	✓	✓	X	X	✓	✓	X	X	X
Franco, E., et al. (2013) [48]	Italy	✓	X	X	✓	X	✓	X	X	X	✓
Garcia-Doval *et al.* (2010) [49]	Spain	✓	X	X	X	✓	✓	X	X	X	X
Gater, A., et al. (2014) [50]	UK	✓	X	X	X	X	✓	X	X	X	✓
Gauthier *et al.* (2009) [28]	UK	✓	X	✓	✓	X	✓	✓	X	X	✓
Gialloreti *et al.* (2010) [29]	Italy	✓	X	✓	✓	X	✓	✓	✓	✓	X
Gil *et al.* (2004) [51]	Spain	✓	X	X	X	X	✓	✓	X	X	X
Gil *et al.* (2009) [52]	Spain	✓	X	X	X	X	✓	✓	X	X	X
Gil-Prieto *et al.* (2011) [53]	Spain	✓	X	X	X	X	✓	✓	X	X	X
Gil-Prieto, R., et al. (2014) [54]	Spain	✓	X	X	X	X	✓	X	X	X	X
Gonzalez *et al.* (2010) [55]	France	✓	X	X	X	X	✓	X	X	X	X
Lionis *et al.* (2011) [56]	Greece	✓	X	✓	X	X	X	X	X	X	✓
Loncar, Z., et al. (2013) [57]	Croatia	✓	X	X	X	X	✓	X	X	X	✓
Lukas *et al.* (2012) [58]	Germany, Spain, Portugal, the Netherlands, Belgium, Sweden and Switzerland	✓	✓	X	✓	X	X	X	✓	X	✓
Mesquita, M. and F. Froes (2013) [59]	Portugal	✓	X	X	X	X	✓	X	X	X	X
Mick *et al.* (2010) [30]	France	X	✓	X	✓	X	✓	✓	✓	✓	X
Moore *et al.* (2010) [60]	UK	X	✓	✓	✓	X	X	X	✓	X	X
Morant-Talamante, N., et al. (2013) [61]	Spain	✓	X	X	X	X	✓	X	X	X	X
Mordarski *et al.* (2009) [62]	Poland	X	X	X	X	✓	X	X	X	X	✓
Nilsson, J., et al. (2013) [63]	Sweden	X	✓	X	X	X	X	X	✓	✓	X
Pierik *et al.* (2012) [64]	Holland/the Netherlands	✓	X	X	X	X	✓	X	X	X	X
Rabaud, C., et al. (2013) [65]	France	✓	X	✓	✓	X	✓	X	X	X	✓
Rehm *et al.* (2010) [66]	14 European countries (countries not specified)	X	X	✓	X	X	X	X	X	X	✓
Schiffner-Rohe *et al.* (2011) [67]	Germany	✓	X	✓	X	X	✓	✓	X	✓	X
Scott *et al.* (2003) [68]	UK	✓	X	X	✓	X	X	X	X	X	✓
Scott *et al.* (2006) [7]	UK	X	✓	X	✓	X	X	X	✓	✓	✓
Serpell, M., et al. (2014) [14]	UK	X	X	X	✓	X	✓	X	X	X	✓
Sicras-Mainar *et al.* (2012) [69]	Spain	✓	✓	X	X	✓	✓	✓	✓	✓	X
Souliotis, K., et al. (2013) [70]	Greece	X	✓	X	X	X	X	✓	✓	X	X
Studahl, M., et al. (2013) [71]	Sweden	✓	X	X	X	X	✓	X	X	X	X
Szucs (2011) [72]	Switzerland	✓	X	X	✓	✓	X	✓	✓	✓	X
Ultsch *et al.* (2011) [73]	Germany	✓	X	X	✓	X	✓	X	X	X	X
Ultsch *et al.* (2012) [31]	Germany	✓	X	X	✓	X	✓	✓	✓	✓	X
Van Hoek *et al.* (2009) [74]	England and Wales	✓	X	X	✓	X	✓	✓	X	X	X
Van Seventer *et al.* (2006) [25]	France, Germany, Italy, Netherlands, Spain, UK	X	X	X	✓	X	✓	X	X	X	✓
Volpi *et al.* (2007) [75]	Italy	✓	X	X	X	X	X	X	X	X	✓
Volpi *et al.* (2008) [76]	Italy	✓	✓	X	✓	✓	X	X	X	X	✓
Weinke *et al.* (2010) [77]	Germany	✓	X	X	✓	X	X	X	✓	X	✓

✓ and x indicate the presence/absence of evidence, respectively.

The references selected for review presented data from 14 European countries with the majority (n = 39, 73.6%) of articles providing data from the ‘Big 5’ EU countries (UK; Spain; France; Germany & Italy). Of the included references, 39 reported data specifically attributed to HZ patients and 35 reported data specifically attributed to PHN patients. Data attributed to a combined HZ and PHN population was included in 15 references. The references eligible for inclusion in the study were all published between 2000 and 2014.

Data presented in reviewed references was categorized as follows: 1) Healthcare utilisation in terms of rates of medical visits, hospitalisations and medication prescribed, 2) Direct costs corresponding to the management of HZ/PHN, 3) Indirect costs, 4) Total costs and 5) Health related quality of life and humanistic burden. Of note, some references contained data pertaining to multiple categories.

### Healthcare utilisation

Information regarding healthcare resource was included in 38 of the identified references (as summarized in Table 3). Data indicated that HZ and PHN are associated with considerable healthcare utilisation, with increased resource use associated with increasing age and the presence of PHN. Data concerning healthcare utilisation was presented in terms of rates of medical visits (including GP and specialist visits), hospitalisations (including duration of stay), use of medications and additional diagnostic/laboratory procedures.

### Medical visits

The majority of patients presenting with HZ will consult their GP at least once [43,50]. Reflecting the chronic nature of PHN, higher rates of medical visits (GP and specialist visits) have been reported in PHN patients compared to HZ patients [14,21,27,29,50]. For example, reports from a study in Italy indicated that patients over the age of 50 with HZ made approximately 1.9 visits to their GP. By contrast, the reported number of visits among patients with PHN was 11.9 and 12.0 (PHN-1 month and PHN 3-months definitions, respectively) [29]. The proportion of patients referred to specialists (74% PHN versus 18% HZ) and the number of specialist visits per case (3.5 and 3.9 for PHN-1 month and PHN 3-months vs 0.2 for HZ) were also higher for PHN patients compared to HZ patients. More frequent specialist visits have also been reported among patients with HZO compared to those with HZ [21].

The average annual incidence of herpes zoster GP-consultations has been reported to increase with age from 32.8/10,000 among those aged <60 years, to 93.1 and 113.2/10,000 among those aged 60–64 years and ≥65 years respectively [64]. Similarly, the average number of specialist visits was also higher in patients over the age of 50 compared to those over the age of 14 [27].

### Hospitalisations

The rates of hospitalisations due to HZ and/or PHN reported in the literature (generally derived from national or insurance databases of hospital admissions) suggest that increased rates of hospitalisation are also associated with increasing age [55,71]. More frequent hospitalisations have also been observed among patients with PHN when compared to patients with HZ across studies in Italy [29], Belgium [40], France [55], Germany [73], Spain [37,45,49,51,52,61]. and Portugal [59]. There are also reports of a greater rate of hospitalisations among female HZ patients compared to male HZ patients at across all age groups [71].

The average length of inpatient stay for PHN (10.2 ± 8.6 days) has been reported to be higher than those associated with HZ (7.8 ± 5.4 days) [29]. The average duration of stay in hospital among HZ patients also appears to be influenced by age [47,74]. For example, analysis of Hospital Episode Statistics (2002–2005) from England indicates the average length of stay among HZ/PHN patients aged 60–64 is 9.3 days whereas those aged 85–90 is 22.3 days [74]. Comparisons between HZ and HZO patients over 50 years of age also indicate longer hospital stays in patients with HZO (9.8 vs 7.8 days) [38].

### Medication use

It is reported that up to 100% of PHN patients and 83.2% of patients with HZ receive medication for their condition across all ages [31]. Polypharmacy is common among HZ patients with HZ patients reported to be taking at least 4 different medications on average and PHN patients reported to be taking 5 medications [50]. This could potentially have wider implications as polypharmacy is known to be a major cause of drug interactions and issues with treatment adherence and safety [78].

The types of medication used has been reported to vary depending on the population being considered (HZ or PHN) [14,21,29,50]. Indeed, current treatment of HZ aims to relieve pain, to limit the spread and duration of the dermatomal lesions and (when started within 72 hours of rash onset) to prevent or alleviate complications associated with HZ. By contrast, for PHN, the focus of medications is to alleviate pain [79,80].

The amount of available information regarding medication use in EU countries was somewhat limited; however available evidence from the literature suggests that, despite the availability of European Guidelines for the management of HZ [80,81], medication prescribing practices (for the treatment of PHN in particular) are not consistent across countries [14,21,25,29,44,50]. An observational study of PHN patients over the age of 50 conducted in France, Germany, Italy, Netherlands, Spain and the UK (n = 84), for example, reported that neuropathic pain medications (89%), analgesics (64%) and anticonvulsants (52%) were most commonly prescribed for the treatment of PHN [25]. However, a study conducted in the UK reported that antidepressants were most commonly prescribed for PHN followed by level 1 and 2 analgesics (59.2%, 55.3% and 50% respectively) [14]. Similarly, studies in Italy and Portugal have reported that anticonvulsants to be the most commonly prescribed treatment for PHN, followed by opioid analgesics [29,44]. Where reported, medication usage for the treatment of HZ was consistent across studies; with antivirals (typically taken for 7 days) the most commonly prescribed treatment [14,21,29,42,50,71].

Of note, despite the widespread use of pharmacological therapies for the management of HZ and PHN, reports from patients indicate dissatisfaction with treatments, particularly in regards to perceived efficacy [14,50].

### Diagnostic/laboratory procedures

Details of additional investigations among patients with HZ and PHN were reported from studies in France, Italy, Spain and the UK [14,21,27,29,50]. Additional investigations reported included blood and urine tests, assessments of inflammation (e.g. erythrocyte sedimentation rate, C-reactive protein level), ophthalmological/ontological examinations, radiology, X-ray, electrocardiograms and ultrasound.

Further diagnostic or laboratory examinations were reported in 28.0% of incident cases of HZ among an Italian sample of immunocompetent individuals, with the mean number of procedures and examinations reported to be 2.6 per case [29]. By comparison, rates of additional investigations reported in France, Spain and the UK were lower [14,21,27,50], with evidence suggesting that rates of additional investigations were higher in those with PHN (10–12.5% compared to HZ (4.4-9%) [14,21,50]. Additional investigations are also more likely among those patients with HZO compared to HZ [21].

### Direct costs of management

Direct costs of the management of HZ and PHN patients were reported in 20 studies. The reviewed articles presented data from Spain [27,37,45,51-53,69], the UK [7,28,43,47,74], France [30], Italy [29,46], Germany [31,67], Belgium [26,40], Greece [70] and Switzerland [72]. Outpatient costs (including medical visits, diagnostic tests and medications), hospitalization and inpatient costs were identified in the literature as key contributors to the overall cost burden of HZ and PHN.

### Outpatient costs

Studies in Belgium [26], Germany [31,67], Italy [29] and the UK [28] have consistently reported higher outpatient management costs (including medical visits, diagnostic tests and medications) among patients who go on to develop PHN. For example, mean direct costs of outpatient management have been reported as €107.98 per HZ episode and €406.04 and €485.51 per PHN episode (1- and 3-month definition respectively) [28]^a^. In addition, costs for outpatient management of HZ and PHN have been reported to increase markedly with pain severity [26,28]. For example, outpatient costs among HZ patients ranging from €85.89 (no pain) to €178.32 (severe pain) per case have been reported in the UK. Reported differences are more pronounced among PHN patients with costs per case ranging from €237.90/284.80 (PHN1/PHN3) among patients with mild pain to €741.91/878.88 (PHN1/PHN3) among patients with severe pain [28]^a^. Key differences in the attribution of outpatient costs for HZ and PHN have been reported for Italy; for patients that did not develop PHN, the majority of the cost of management has been reported to be due to the cost of medication (83%) whereas for patients that develop PHN a greater proportion of cost was attributed to diagnostic and investigative procedures [29]. Other studies, however, indicate costs of diagnostic procedures to be the smallest contributor to outpatient costs among patients with HZ and PHN (PHN-1 and PHN-3) [27].

While the average cost of outpatient management per case is higher for patients with PHN than for patients with HZ, when considering overall burden of disease at a country level, the overall cost of disease is higher for HZ. This is primarily a result of the higher incidence rates of HZ. These differences were highlighted by one study which investigated the annual cost of HZ and PHN to healthcare providers located in England and Wales [47]. Based on an estimated 260,995 episodes of HZ occurring annually, with 14% of cases developing PHN, an estimated €76.39 million^a^ is spent on the management of HZ (including PHN). Of this, €52.32 million was attributable to HZ (€22.47 m due to hospitalisation, €19.26 m due to prescription medications and €9.63 m due to GP consultations) and €24.07 million to PHN1 (cost breakdown not provided). Similar findings are reported in Germany where statutory health insurance bills have been estimated at €115.5 m; €93.5 m attributed to HZ treatment and €22 m attributed to treatment of PHN [31].

### Hospitalisation and inpatient costs

Hospitalisation costs contribute significantly to the overall direct cost of managing HZ and PHN. Rates of hospitalisation and duration of stay were found to be higher for PHN than for HZ, and to increase with increasing age, translating into higher inpatient costs for patients that develop PHN (Table 4) [18,24,33,70]. Note that data on hospitalisations and inpatient costs was primarily extracted from a range of national and insurance databases in each country. No studies identified provided a breakdown of the cost elements that are included in a ‘hospital stay’.

**Table 4:** **Mean cost per hospitalised case of HZ/PHN, by country**

**Country**	**HZ/PHN**	**HZ (without PHN)**	**PHN**
Belgium [40]	-	€5,982 (€798-31,689)	-
		(all ages)	
France [41]	-	€2,774.28 (50+years)	€3,731.09 (50+years)
Germany [31]	-	€4,189.74 (50+years)	€4,279.68 (50+years)
		€3,282.64 (all ages)	€4,112.03 (all ages)
Italy [29]	-	€3,188.16 ± €1,614.99 (50+years)	€3,451.38 ± €3,248.43 (50+years)
Spain [37,45,52,53]	€3,366.4 -4,505.6	€5,019.84 (aged 40–49)	€5,108.04 (aged 50–59)
	(all ages)	€4,495.68 – 5,139.54 (aged 50–59)	€4,732.56 (aged 60–69)
		€4,210.92 – 4,782.96 (aged 60–69)	€4,478.04 (70+)
		€4,062.24 – 4,556.16 (aged 70+)	

### Indirect costs

Indirect costs considered in the EU literature were mainly related to costs associated with sick leave (absenteeism). Seventeen articles reported data on sick leave due to HZ and PHN and the costs that this incurs [7,26,27,29-31,40,42,58,60,63,69,70,72,73,77,82]. Absenteeism due to HZ and PHN is a significant cost driver, with increased costs found to be associated with the presence of PHN [29,31,72] and increased levels of pain experienced [26,72]. Up to 65% of employed patients report work absence due to their disease [77].

Costs associated with absenteeism varied across EU countries. In France, these costs accounted for up to 30% of the costs of management of HZ from the societal perspective and up to 10% of the costs of management for PHN in patients aged 50 years and over [30]. In Germany, the mean annual cost of sick leave due to HZ was €1,504 per patient (increasing to €1,826 for patients over the age of 50), with patients missing an average of 12.5 days from work. The costs associated with PHN were found to be higher at €7,183 (€7.590 for patients over 50) with patients missing an average of 2 months from work [31]. The average costs associated with absenteeism were found to be lower in Italy and Switzerland than those reported for Germany [29,72]. Average costs for patients over the age of 50 in Italy were €684 per case of HZ and €978 per case of PHN [29]. In Switzerland, reported costs of sick leave per case for patients with varying levels of pain were as follows: mild pain (HZ €335; PHN 381), moderate pain (HZ €421; PHN €1,018), severe pain (HZ €1,837; PHN €2,443)^b^ [72].

In Spain, the findings from an observational study [27] of 130 HZ patients aged 19–45 (median age of 63.5 yrs) who were observed for 1 year suggest that an average of 4.3 hours of work were missed per patient at a cost of €52.98. This study also found that a total of 6 work hours were lost by carers across the study sample of 130, resulting in a total loss of €86.31. The cost and time implications of time taken off work by carers were also considered in two further studies that were conducted in Belgium and the UK respectively. The study conducted in Belgium found that 14/184 patients reported that someone else could not go to work due to their illness for between 0.5-10days, resulting in an average loss of €52 per case due to carer absenteeism [40]. For the study conducted in the UK, among 70 HZ patients, a total of 52 work days were lost by their carers [7]. However as the majority of the reviewed studies usually do not report the cost burden associated with time lost by carers this cost is likely to be underestimated.

### Total costs of management

A total of 10 studies reported the total cost per case of HZ and/or PHN in EU countries, taking into account both direct costs (including medical visits, medications and hospitalisations) and indirect costs (including time missed from work). Table 5 presents the average cost per case in each country based on the data that was detailed in the reviewed literature. Overall, higher costs were consistently associated with the presence of PHN and increased levels of pain. Of note, whilst HZ and PHN are associated with higher costs to healthcare providers in elderly populations, costs to society in terms of time missed for work tends to be lower, so the total costs overall are similar across age groups [60].

**Table 5:** **Mean total cost of management per HZ or PHN case**

**Country**	**Population age**	**Cost per case**		
		**HZ**	PHN1*	**PHN3^**
Belgium [26]	Aged ≥50	€118 (no pain)	€156.94 (mild pain)	€329.22 (mild pain)
		€1,150.50 (severe pain)	€1,085.60 (severe pain)	€2,037.86 (severe pain)
France [30]	Aged ≥50	€419.87 (271–586)	€672.76 (451–968)	-
Germany [31]	Aged ≥50	€388.30 (354–425)	-	€1,427.80 (1,002-1,917)
Germany [67]	Aged ≥50	€729.57	€ 2,167.89	
Italy [29]	Aged ≥50	€888.06	-	€1,66.65
Spain [27]	Aged >14	**Overall:** €446.04 (SD 508.58)	€647.82 (SD 684.4)	€968.78 (SD 803.58)
		**50-59:** €282.06 (SD 273.15)		
		**60-69**:€510.52 (SD 512.73)		
		**>70:** €422.91 (SD 388.66)		
Spain [69]	Aged >30	€460.95	€1,956.15	-
Sweden [63]	All ages		€902.88 (HZ and PHN)	
Switzerland [72]	Aged ≥50	€394.46 (no pain)	-	€381.15 (no pain)
		€1,835.57 (severe pain)		€2,442.99 (severe pain)
UK [7]	Not specified	€793.60 (31–6,394)	-	-
	(all ages)	**Aged +65:** €788.48		

*PHN defined as pain persisting for one month following resolution of HZ rash.

^PHN defined as pain persisting for three months following resolution of HZ rash.

**Figure 2:**
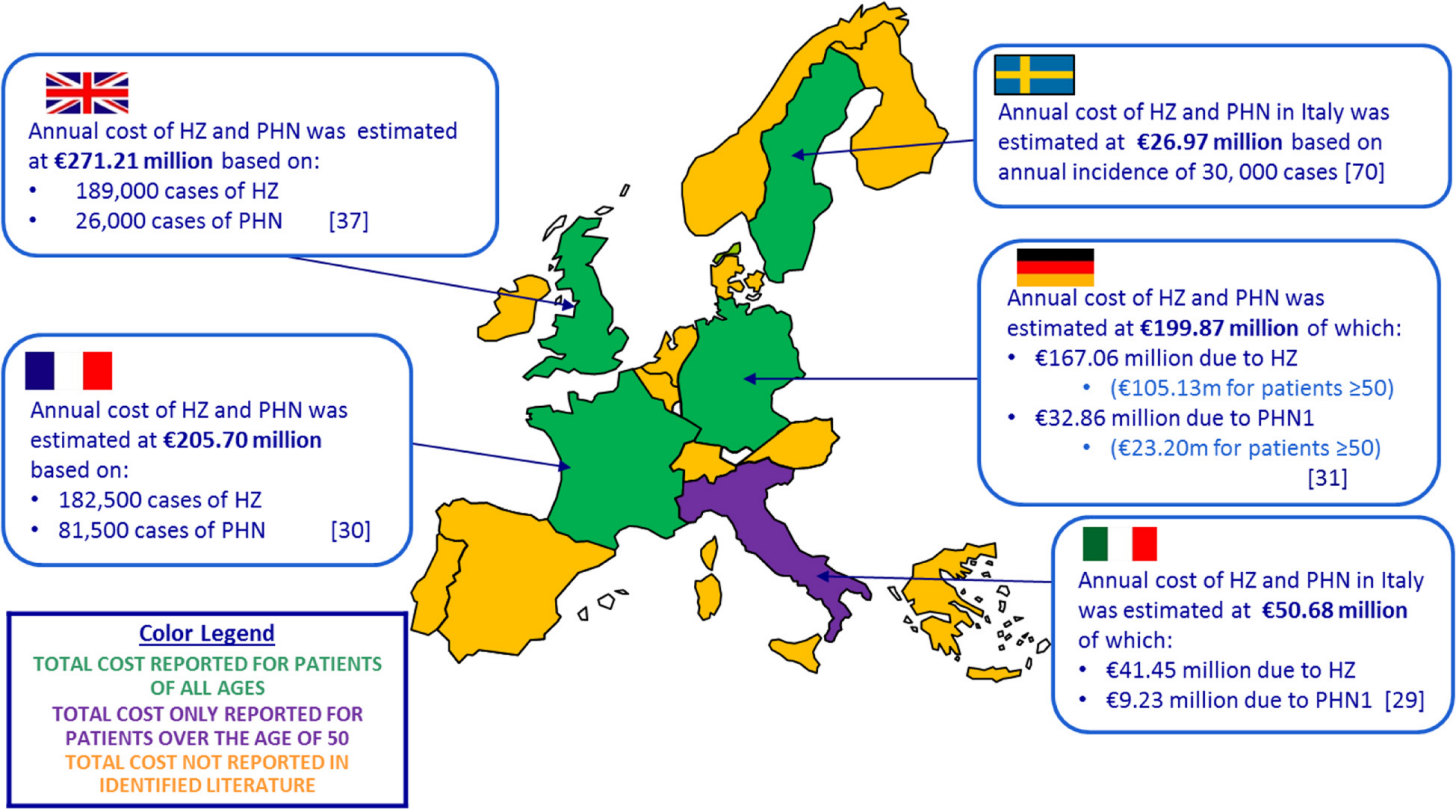
**Estimated total cost burden associated with HZ and PHN considering outpatient, hospitalisation and indirect costs.**

The total annual cost (direct and indirect costs) of HZ and PHN on a country level basis was only available for the UK [43], France [30], Germany [31], Italy [29] and Sweden [63]. Two studies presented data separately for the cost of HZ and PHN and patients over the age of 50 [29,31]. Further details are provided in Figure 2.

### Humanistic burden and impact on quality of life

A total of 21 EU studies [7,14,15,21,25,28,42,44,48,50,56-58,62,65,66,68,75-77] reported data on the humanistic burden associated with HZ and PHN, with the significant negative impact of HZ and PHN on patients’ HRQOL consistently reported. Of note, pain experienced due to the disease was found to interfere with many aspects of patient’s daily life. Greater interference was associated with increased levels of pain [25,83] and the presence of PHN was associated with a greater impact on most domains of HRQoL [14,50,58,77]. Patients also reported consequences for their family and social circle when suffering from HZ or PHN [58,77,84].

### Pain and interference with daily activities

Reports within the reviewed literature indicate that patients with HZ and PHN experience moderate-severe pain. Ratings of average and worst pain reported in the literature are higher among PHN patients compared to HZ patients [14,41,50,58,77].

A number of the reviewed articles provided data from the Zoster Brief Pain Inventory (ZBPI) and the Modified Brief Pain Short Form (mBPI-SF)^c^ – derivative measures of the Brief Pain Inventory (BPI) which require patients to rate the level of interference they experience on a scale of 0–10 (‘does not interfere’ – ‘completely interferes’), across seven health status domain; general activity, mood, walking ability, normal work, social relations, sleep and enjoyment of life. Table 6 presents the mean scores for patients with HZ and PHN from five studies. In general, reports of pain interference on all seven health status domains were greater among patients experiencing PHN compared to patients with HZ [14,50,58,77]. HZ and PHN patients reported sleep to be the aspect of their daily lives most affected by pain (4.5-4.9 and 6.3-6.5 mean scores respectively), while walking ability was the least affected in HZ patients (1.7-4.0 mean score) and enjoyment of life was least affected in PHN patients (3.8-5.2 mean score) [14,15,50,58,77]. Increased levels of pain are reported to be associated with increased levels of interference [25,83]. Levels of pain interference, however, do not appear to be associated with age. Lukas et al. [58] presented pain interference data separately for six European countries included in the study. While differences in the level of interference observed varied from country to country, the domains of health status most affected was largely consistent.

**Table 6:** **Level of pain interference across seven health state domains as assessed by the ZBPI (or similar instrument)**

**Health state domains**	**HZ (mean scores)**	**PHN (mean scores)**
**General activity**	3.8-4.4	3.1-5.7
**Mood**	3.4-4.5	3.4-5.9
**Walking ability**	1.7-4.0	1.7-5.8
**Normal work**	3.3-4.4	2.9-6.1
**Social relations**	2.1-3.5	2.1-5.4
**Sleep**	4.5-4.9	6.3-6.5
**Enjoyment of life**	3.6-4.0	3.8-5.2

Data from Bouhassira 2012 [15], Gater 2014 [50], Lukas 2012 [58], Serpell 2014 [14], Weinke 2010 [77].

### Impact on general health-related quality of life

A number of studies have included generic HRQoL measures such as the SF-12 [15,83], SF-36 [14,21,50], EQ-5D [7,14,25,50] and Short Italian Questionnaire (SIQ) [75,76] to assess HRQoL in HZ and PHN patients. Generally, HRQoL has found to be inversely associated with levels of reported pain [14,25,50,83] and the poorest HRQoL has been observed for patients with PHN [14,21,50].

**Figure 3:**
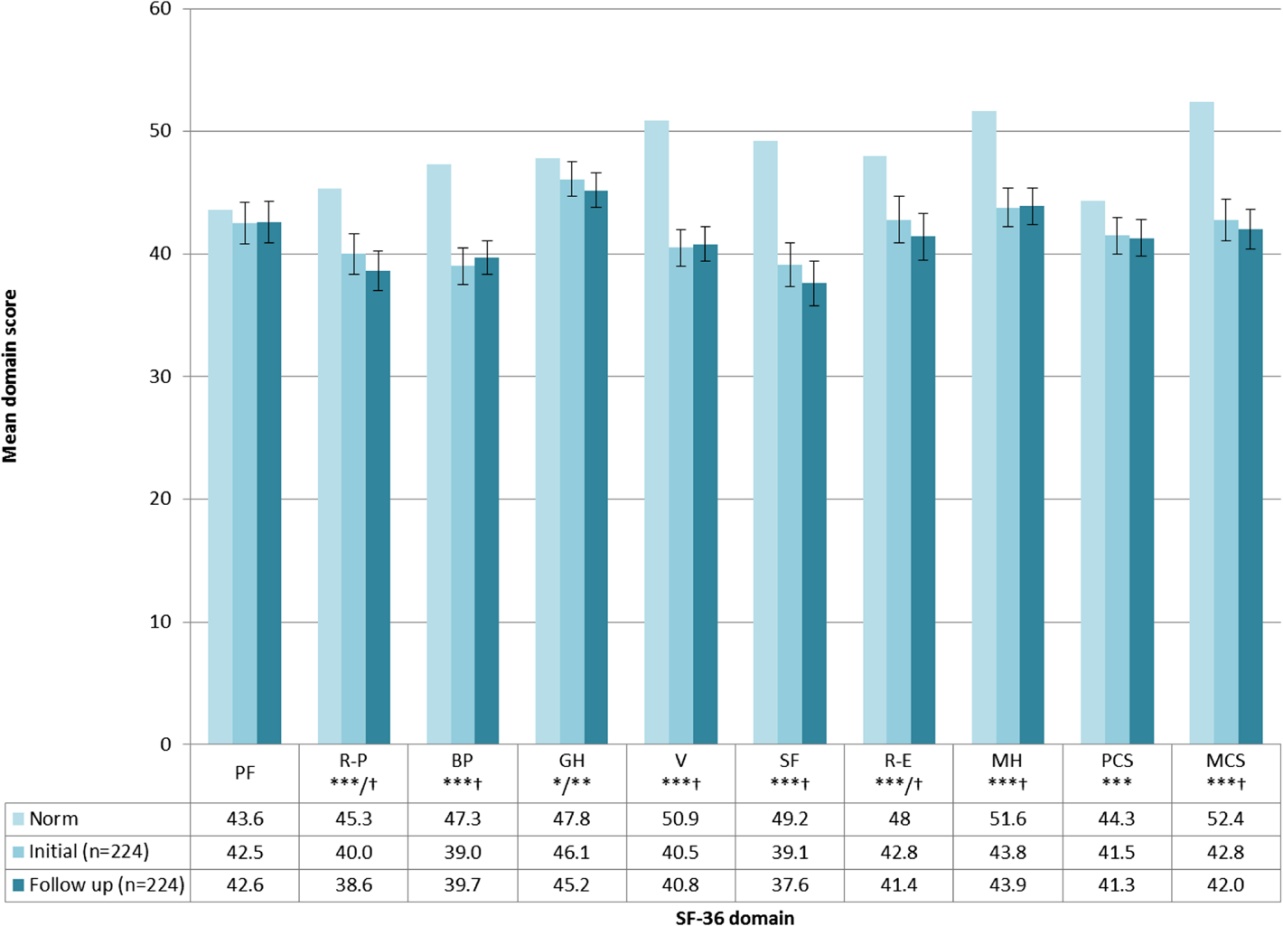
**Comparison of SF-36 domain and component scores for HZ patients compared to age-matched norms [** 50 **].** No *s p≥0.05, *p<0.05; **p>0.01; ***p <0.001. † = clinically meaningful PF=Physical functioning, R-P=Role-physical, BP=Bodily pain, GH=General health, V=Vitality, SF=Social functioning, R-E=Role-emotional, MH=Mental health, PCS=Physical component summary, MCS=Mental component summary. *Note*: Data collected on initial presentation of HZ to healthcare professional and then again 7-14 days later (follow-up).

**Figure 4:**
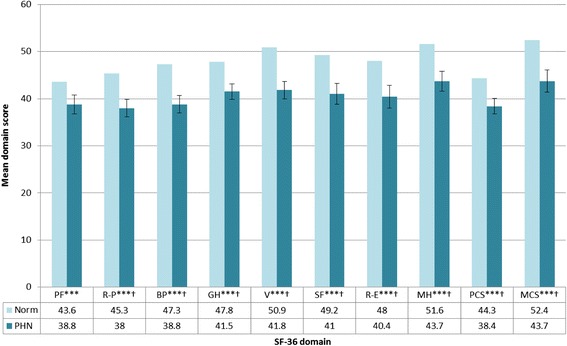
**Comparison of SF-36 domain and component scores for PHN patients compared to age-matched norms [** 14 **].** No *s p≥0.05, *p<0.05; **p<0.01; ***p <0.001. † = clinically meaningful PF=Physical functioning, R-P=Role-physical, BP=Bodily pain, GH=General health, V=Vitality, SF=Social functioning, R-E=Role-emotional, MH=Mental health, PCS=Physical component summary, MCS=Mental component summary.

Consideration of HRQoL scores from normative populations indicates an inverse relationship between age and HRQoL. As a condition mainly inflicting the elderly, therefore, HRQoL is already considerably compromised in HZ and PHN patients. Nonetheless, recent research indicates that both HZ and PHN patients demonstrate statistically significant and clinically relevant deficits in HRQoL when scores for validated measures of HRQoL (e.g. SF-36 and EQ-5D) among these populations are compared to those from aged-matched normative samples (Figures 3 and 4) [14,50].

### Impact on mood and mental well-being

Data presented across studies demonstrates that patients experience a negative impact on their mood and mental well-being, with patients reporting stress, anxiety and depressive symptoms [14,15,50,56,58,76,77]. Across studies, a range of instruments have been used to assess the impact of HZ and PHN on mood, including the Hospital Anxiety and Depression scale (HADS) [7,15], 10,47 Short Italian Questionnaire (SIQ) [75,76], the Short Anxiety Screening Test (SAST) [56] and bespoke questions [58,77]. Up to 29% of patients with HZ reported feelings of moderate anxiety or depression, rising to 43% in patients with PHN. Further, a larger percentage of PHN patients reported extreme anxiety or depression (11% vs. 5%) [58,77], evidencing that mood and mental well-being issues are greater for PHN patients. The majority of patients reported experiencing stress some of the time (up to 78% for HZ and 91% for PHN) or most of the time (up to 19% HZ and 41% PHN) [58,77].

### Impact on family and social relations

Up to 50% of patients with HZ and 81% of patients with PHN reported consequences for their family members and social circle [58,77]. Despite the high proportion of patients reporting impacts on their social and family lives, only three studies assessed this issue beyond comparing scores on social dimensions of instruments such as the ZBPI or SF-36 [58,77,84]. In one study in particular, one fifth of the respondents felt isolated from their family and friends while suffering from HZ or PHN, with 19% and 27% of patients respectively reporting reduced communication during their illness [58]. Of note, impacts were not limited to the patient themselves, with findings from a qualitative focus group of patient’s relatives demonstrating that relatives were worried and stressed by the impact of HZ on the patients [84].

## Discussion

The aim of this article was to provide a holistic overview of the published literature concerning the humanistic, economic and societal burden of HZ and PHN in Europe. The reviewed evidence highlights the significant burden of HZ and PHN for patients, healthcare systems and wider society. A number of key findings are noted. Firstly, the direct costs of managing HZ represent a significant proportion of the total costs associated with the disease, with higher costs associated with the presence of PHN and with greater pain severity. Secondly, absenteeism was identified as a significant cost driver (albeit in a small number of studies), with up to 65% of employed patients reporting work absence due to their disease. Lastly, research suggests that age, pain severity and the presence of PHN are the key drivers of burden to patients, healthcare systems and society.

In Europe the percentage of people aged 65 years or over is projected to increase from 17.1% (84.6million) in 2008 to 30.0% (151.5 million) in 2060 [85]. This demographic shift is likely to have a profound influence on the incidence of HZ and PHN and may contribute to increased concern among European healthcare authorities; particularly if economic crisis and healthcare budget constrains persist in the long term. Findings from this review indicate that the direct costs of HZ and PHN management are greater for older patients. In addition, older adults typically have lower HRQoL than younger adults (deficits which are more pronounced with the presence of HZ and PHN) and may be less able to complete activities of daily living and self-care unassisted. The potential implications of an aging population to wider society, therefore, are wide reaching.

### Limitations of the current literature and potential gaps that provide opportunities for future research

A number of limitations concerning the currently available body of evidence were identified.

### Inconsistencies in data

There is currently no consensus regarding the definition of PHN and a lack of consistency in the definition of PHN employed in studies was evident throughout the review. This presents a number of challenges when attempting to accurately understand the burden of HZ and PHN. For example, it has been demonstrated that the prevalence of PHN varies dramatically depending on the point at which HZ-related pain becomes classified as PHN (PHN1 or PHN3) [86]. Accurately attributing costs to HZ and PHN and determining the overall cost burden of the conditions, therefore, is difficult. Similarly, cost per case may vary dramatically depending on the definition employed. For this reason, PHN definitions employed in the referenced studies have been highlighted throughout this review and should be considered when interpreting the findings from individual studies.

Furthermore, there is a lack of consistency in the use of International Classification of Diseases (ICD) codes used to identify PHN patients in clinical practice. As a result, there may be a significant proportion of PHN patients who have not been considered in these studies; consequently decreasing confidence in the accuracy of estimates regarding the burden of PHN.

A lack of consistency in the ways in which data had been collected provides further difficulties in interpreting and comparing the available data across studies, with databases, patient records and patient surveys all utilized. For example, rates of resource use were derived from a range of sources including: analysis of patient records or an observed sample, patient responses to surveys and national and insurance databases, with each of these methods having their own limitations. The use of databases is a valuable way of gathering data at a national level, however it assumes information is recorded consistently and accurately across databases and that the database has adequate and representative population coverage. Analysis of patient records assumes information is accurately recorded by GPs, who have a myriad of demands on their time, and analysis of patient’s responses assumes accuracy in their recall. Of note, the incidence of hospitalisations were largely based on rates derived from databases in different countries, or rates recorded in observed samples, thus inaccuracies may occur when these data are extrapolated to the country population. Variation in reports of healthcare resource use, indirect costs and humanistic burden are also likely to have resulted from inconsistency in the time frame that different studies considered relevant.

With respect to the literature on economic burden, it is understood that costs are likely to vary between countries in terms of the cost of medical resources, medical visits and prescribed medications. Currently these differences are not reflected in the published literature and in some cases, the cost of treating a case of HZ or PHN were calculated inconsistently, with some studies presenting the cost per treated case while others presented the average across all patients. As such there was a wide variation between studies. Only one study provided costs (per treated case) for the different types of medications used to treat HZ or PHN [46]. Similarly, no studies provided breakdown or clarification of cost elements included in a ‘hospital stay’. Great caution, therefore, should be taken in comparing and interpreting the available data. Furthermore this highlights an unmet need for data that clearly distinguishes between HZ and PHN and also for ‘per treated case’ data for a wide range of EU countries.

A further notable limitation was that data were not available for all EU countries. Despite comprehensive database searches, in which no restrictions on country of interest or language of publication were implemented, cost burden data for the majority of Eastern European countries was not identified. The majority of studies, instead, focus primarily on the UK, Spain, France, Italy, Belgium and Germany.

### Gaps in evidence

A number of evidence gaps were identified, some of which provide opportunities for future research. A key gap identified in the available evidence pool relates to how indirect costs are assessed. While a number of EU studies presented data on the time missed from work and associated costs, data on productivity while at work and associated costs is lacking. Studies in the US and Canada, however, report that presenteeism could account for up to 75% of the cost burden associated with work loss [87] and that an average of 46 hours were lost (per employed patient) due to presenteeism [88].

In Europe, decisions regarding access and reimbursement of medical interventions are predominately driven by considerations of cost-effectiveness. Consideration of the impact of conditions as reported by patients themselves, however, provides important information regarding unmet need and the efficacy of medical interventions. A number of studies assessing aspects of humanistic burden associated with HZ and PHN were identified. However, there are number of notable limitations. Firstly, the impact of HZ and PHN was generally assessed using disease specific questionnaires (such as the ZBPI) or ‘bespoke’ questions which have not been tested or validated. There was only limited use of generic HRQoL instruments in the EU studies conducted to date. However, data from such instruments are valuable for quantifying burden with reference to the normative population and patients with other diseases – data that is important for contextualizing the relative burden associated with HZ and PHN. The available evidence does not typically look beyond the pain which accompanies or persists beyond the rash and the omission of other factors that can impact on patients’ quality of life (e.g. fatigue, stomach upsets, allodynia, and numbness). This should be addressed, preferably through a combination of quantitative and qualitative research, the latter of which was notably lacking in the literature.

Finally, the lack of literature relating to the wider societal burden potentially caused by HZ and PHN (particularly with respect to the impact on caregivers and the broader implications of HZ and PHN on provision of formal and informal social care) may be considered a critical gap. To date burden among informal caregivers has rarely been considered, and only in terms of time lost from work. Significantly, this omits the potential impacts of providing informal care to patients that may not have resulted in time lost from work (e.g. negative impact on the health and well-being of caregivers). Furthermore, consideration of healthcare resource use and the economic burden of HZ and PHN has typically focussed on impacts within the healthcare system; the broader implications of HZ and PHN are rarely appreciated. For example, the functional deficits observed among HZ and PHN patients that may lead to a loss of autonomy and increased reliance on formal paid care have not been estimated. Beyond some evidence of direct and indirect costs related to the disease, the potential impact of HZ and PHN on the social care system remains unknown.

## Conclusions

This literature review highlights the burden faced by patients, healthcare systems and society due to HZ and PHN in Europe. While the incidence of HZ and PHN increases with age, the total cost per case of HZ and PHN management is not necessarily age-dependent. Pain severity was associated with increased costs for both HZ and PHN, and, as a chronic condition, costs were higher for patients experiencing PHN. No study fully addressed the humanistic and economic burden of HZ and PHN to patients, their families and society as a whole. Based on the reviewed evidence and an assessment of the gaps in the evidence base, it is likely that the true burden of HZ and PHN is underestimated in the Europe. Further research is required to build a holistic understanding of the impact of HZ and PHN to patients, healthcare systems and society.

### Endnotes

^a^*Costs originally reported in 2006 GBP sterling (£), converted to euros (€) using the following conversion rate £1 = €1.18.

^b^*Costs originally reported in 2006 Swiss francs (CHF), converted to euros (€) using the following conversion rate 1CHF = €0.81.

^c^Although 2 studies do not report a specific instrument, items reported appear to be derived from the ZBPI (38, 55).
